# Lung adenocarcinoma with an uncommon *CCDC85A‐ALK* fusion responding to alectinib: A case report

**DOI:** 10.1111/jcmm.17520

**Published:** 2022-09-14

**Authors:** Jieheng Lin, Wenping Wang, Jietao Lin, Ruilian Chen, Yang Cao

**Affiliations:** ^1^ The First Affiliated Hospital of Guangzhou University of Chinese Medicine Guangzhou China

**Keywords:** Alectinib, ALK kinase inhibitors, *CCDC85A‐ALK*, fusion, lung adenocarcinoma

## Abstract

A 55‐year‐old Chinese man with a right lung mass and lymph node metastasis (T4N3M0 IIIB) was diagnosed with lung adenocarcinoma after a CT‐guided biopsy. With the wide application of next‐generation sequencing (NGS) in tumour detection, we found a rare *CCDC85A‐ALK* fusion. The patient received alectinib, which had marked efficacy. This is the first report of a lung adenocarcinoma patient harbouring a new uncommon anaplastic lymphocyte kinase fusion that showed a remarkable response to alectinib. NGS aids in selecting treatment in non‐small cell lung cancer patients.

## 1 INTRODUCTION

Lung cancer is one of the leading causes of cancer‐related mortality worldwide.^1^ Anaplastic lymphocyte kinase (ALK) rearrangements are driver genes in non‐small cell lung cancer (NSCLC), accounting for 2%–7% of NSCLC. *EML4‐ALK* is the most common ALK gene arrangement, and some non‐canonical ALK fusions have been reported.^2^, ^3^ For advanced ALK‐positive NSCLC, alectinib is recommended as first‐line treatment. No differences were observed in the results of different EML4‐ALK variants with alectinib in the ALEX trial; however, further studies are needed to confirm the efficacy of alectinib in other non‐canonical ALK fusions compared with that in EML4‐ALK.

This is the first report of a lung adenocarcinoma patient harbouring a *CCDC85A‐ALK* rearrangement that showed a remarkable response to alectinib. This case suggests that alectinib is an option for NSCLC patients with uncommon ALK gene rearrangements.

## 2 CASE REPORT

**FIGURE 1:**
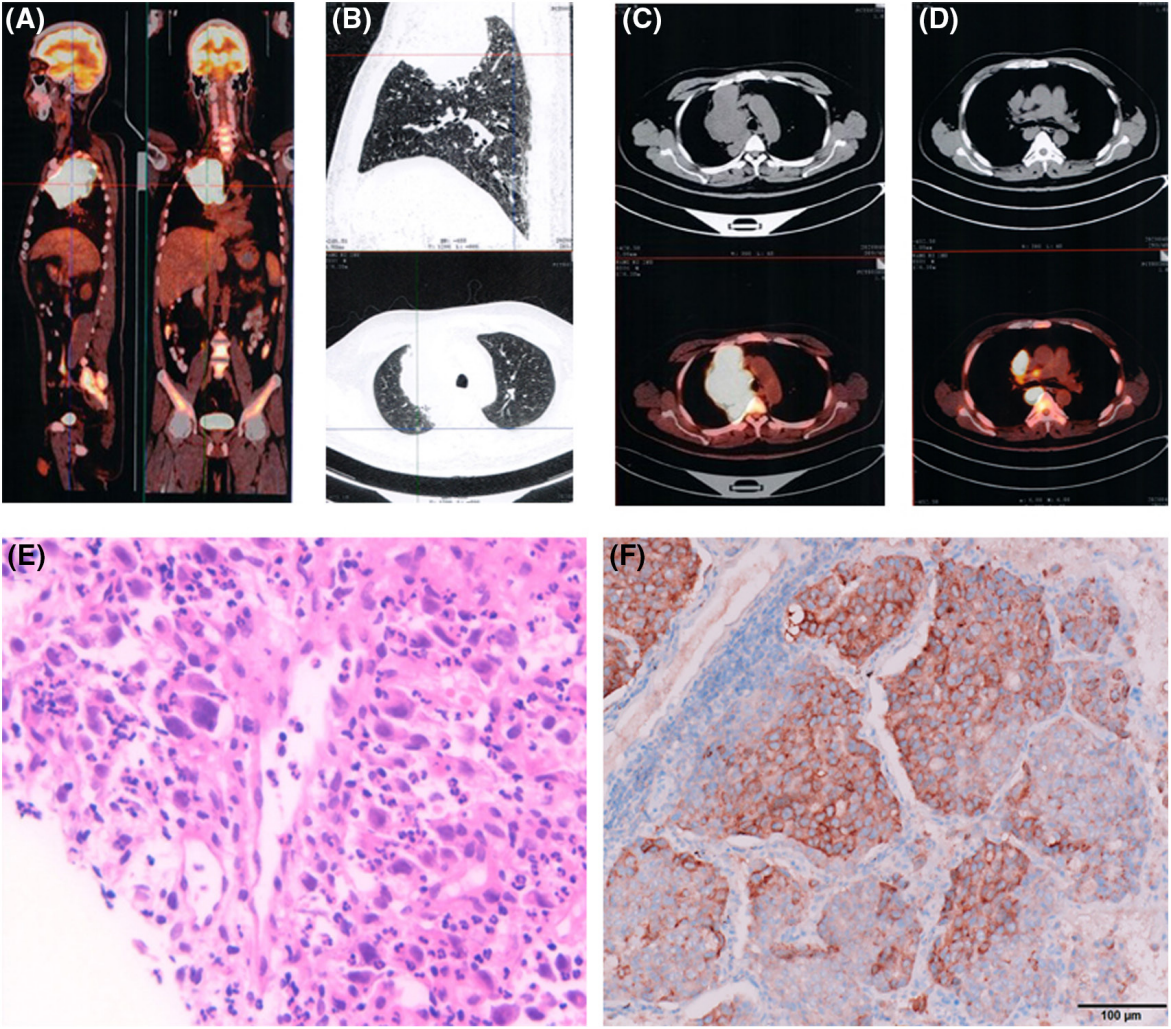
(A–D) PET/CT showed that there were large hypermetabolic lesions in the right mediastinum, which straddled the anterior, middle and posterior mediastinum, and protruded into the lung field and involved the adjacent pleura. (E) The histology of adenocarcinoma with poor differentiation. (F) ALK (+) IHC using the D5F3 ALK antibody.

A Chinese man was hospitalized in April 2020 with an irritating dry cough and chest pain for more than 1 month. PET/CT showed a large hypermetabolic lesion (ca. 76 × 92 × 97 mm^3^) in the right mediastinum, straddling the anterior, middle and posterior mediastinum and protruding into the lung field and involving the adjacent pleura. In the mediastinum (4R group), the lymph nodes were enlarged and metabolically active. Lymph node metastasis was suspected (Figure 1). Under CT guidance, a lung biopsy was performed. The pathological diagnosis was confirmed as poorly differentiated lung adenocarcinoma (Figure 1). The immunohistochemistry was CD30(−), CK(partial+), TTF‐1(focal+), P40(−), CK7(−), vimentin(+), CD7(−), calretinin(−), Ki‐67(70% +), CK(35BH11) (focal+), CgA(−), CK8/18(partial+), Syn(−) and ALK(D5F3)(+).

**FIGURE 2:**
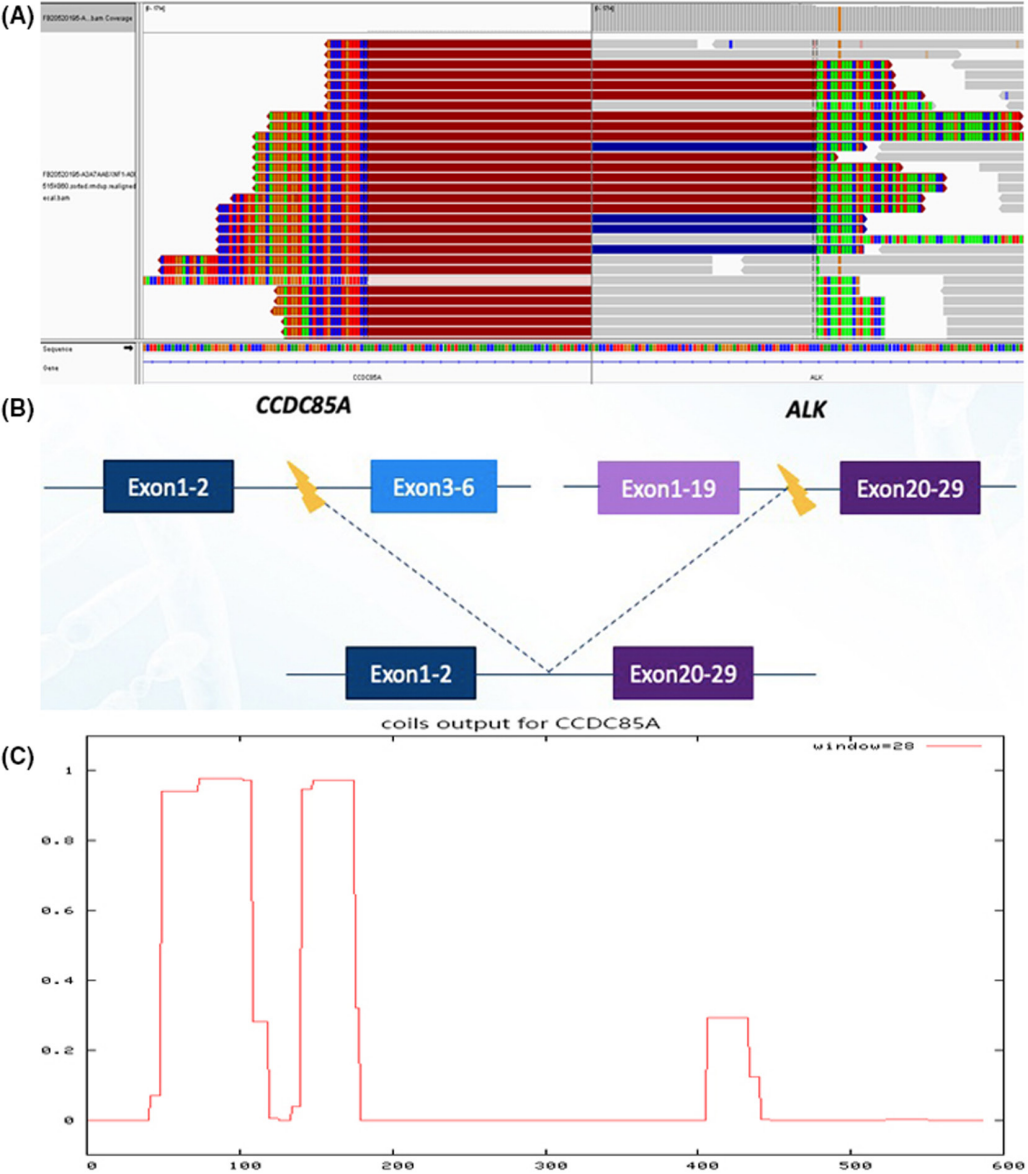
(A) Integrative Genomics Viewer snapshot of CCDC85A‐ALK by next‐generation sequencing (NGS). (B) A schematic representation of the CCDC85A‐ALK fusion. Exons 1‐2 (1‐413aa) region of CCDC85A gene and ALK (exon 20), with a hypothetical CCDC85A‐ALK fusion. (C) We used the Coils server (https://embnet.vitalit.ch/software/COILS_form.html) to compare the amino acid sequence of CCDC85A gene to a database of proteins known to form coiled coils and found that the probability of a coiled coil domain at the exons 1‐2 (1‐ 413aa) region of CCDC85A gene being present is almost 100%. The *x*‐axis represents the position in the protein by amino acid number, and the *y*‐axis shows how strongly that region is predicted to form a coiled coil domain. ‘Window’ refers to the width of the amino acid ‘Window’ that is scanned at one time.

To explore potential targeted therapies, next‐generation sequencing (NGS) was performed on the pulmonary biopsy tumour tissue and a blood specimen. This revealed a *CCDC85A‐ALK* fusion variant and missense mutation of exon 8 (p.P540S) (Figure 2). The patient refused radiotherapy and chemotherapy, but agreed to take alectinib (600 mg bid).

**FIGURE 3:**
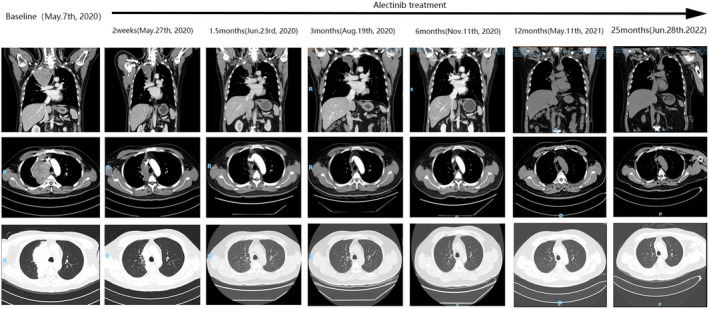
Significant reduction in the tumour volume was observed by the follow‐up CT scans from 2 weeks to 25 months post‐alectinib therapy. The most recent CT scan showed that the patient's lung tumour was close to disappearing, and he had almost achieved a clinical complete response (CCR).

After 2 weeks of alectinib, the tumour was markedly smaller on CT. The patient tolerated the alectinib well, with a sustained partial response (Figure 3). Periodic re‐examination has detected no obvious adverse reactions.

## 3 DISCUSSION

Anaplastic lymphocyte kinase is a transmembrane protein and an insulin receptor tyrosine kinase. Echinoderm microtubule‐associated protein‐like 4 *(EML4)‐ALK* is the most common ALK gene arrangement, and it is found in 3%–13% of NSCLC.^4^ Different ALK fusion genes and *EML4‐ALK* variants have been reported in NSCLC, and they respond to ALK inhibitors differently.^5^

The incidence of uncommon ALK gene rearrangements in ALK‐positive Chinese NSCLC is 18%–19%, and the clinical efficacy of ALK inhibitors needs further study.^2^, ^6^ In this case, a rare gene fusion had a marked clinical response to alectinib, a highly ALK selective inhibitor recommended as a first‐line drug for advanced ALK‐positive NSCLC. The Global Phase III ALEX Study confirmed the superior efficacy of alectinib versus crizotinib in untreated ALK‐positive NSCLC. The median progression‐free survival was 34.8 months with alectinib and 10.9 months with crizotinib.^7^ In our case, the tumour shrank markedly after the patient had taken alectinib for 2 weeks.

In this patient, NGS showed that the ALK gene possessed a *CCDC85A‐ALK* fusion variant due to the rearrangement of exon 2 of *CCDC85A* and exon 20 of ALK. We predicted that the *CCDC85A* gene had a coiled coil domain, which is thought to drive the dimerization of the ALK fusion protein and activate self‐phosphorylation of the ALK kinase domain in the fusion protein, triggering ALK downstream signalling pathways.^8^, ^9^ This is the first discovery of a *CCDC85A‐ALK* rearrangement in a lung adenocarcinoma patient before treatment. We speculated that this fusion constitutively activated kinase participating in tumorigenesis and cancer progression, and increased the sensitivity of the tumour to ALK kinase inhibitors (Figure 2).

This is only one case report, and the long‐term efficacy of alectinib in this patient will require further observation. The lack of in vitro studies of the signal pathway caused by the fusion, and its sensitivity to various ALK kinase inhibitors is also a deficiency of this report.

In summary, this is a successful case of a lung adenocarcinoma patient receiving an ALK kinase inhibitor. This case suggests that the newly found *CCDC85A‐ALK* fusion is sensitive to alectinib. These findings demonstrate the importance of NGS in the diagnosis of and treatment for patients with NSCLC. NGS may identify more rare mutations, provide more comprehensive mutation information for clinicians and NSCLC patients, and help in the selection of appropriate treatments.

## AUTHOR CONTRIBUTIONS

**Jieheng Lin:** Conceptualization (equal); formal analysis (equal); writing – original draft (equal); writing – review and editing (equal). **Wenping Wang:** Data curation (equal); formal analysis (equal). **Jietao Lin:** Data curation (equal); methodology (equal); supervision (equal). **Ruilian Chen:** Data curation (equal); writing – original draft (equal). **Yang Cao:** Conceptualization (equal); data curation (equal); investigation (equal); methodology (equal); resources (equal); validation (equal); writing – review and editing (equal).

## CONFLICT OF INTEREST

The authors declared that they have no conflicts of interest to this work. We declare that we do not have any commercial or associative interest that represents a conflict of interest in connection with the work submitted.

## CONSENT STATEMENT

The patient had signed informed consent prior to his hospitalization that biological specimens collected during his hospitalization might be used for scientific research.

## Data Availability

Data available on request due to privacy/ethical restrictions.

## References

[1] Siegel Rebecca L , Miller Kimberly D , Ahmedin J . Cancer statistics, 2020. CA Cancer J Clin. 2020;70:7‐30. 31912902 10.3322/caac.21590

[2] Jin K , Xu‐Chao Z , Hua‐Jun C , et al. Complex ALK fusions are associated with better prognosis in advanced non‐small cell lung cancer. Front Oncologia. 2020;10:596937. 10.3389/fonc.2020.596937 PMC7759679 33363027

[3] Benjamin S , Marileila V‐G , Ross CD . ALK gene rearrangements: a new therapeutic target in a molecularly defined subset of non‐small cell lung cancer. J Thorac Oncol. 2009;4:1450‐1454. 20009909 10.1097/JTO.0b013e3181c4dedb

[4] Yuan Y , Yu‐Min L , Chung‐Tsen H , et al. Novel targeted therapeutics: inhibitors of MDM2, ALK and PARP. J Hematol Oncol. 2011;4:16. 21504625 10.1186/1756-8722-4-16 PMC3103487

[5] Heuckmann Johannes M , Hyatt B‐W , Florian M , et al. Differential protein stability and ALK inhibitor sensitivity of EML4‐ALK fusion variants. Clin Cancer Res. 2012;18:4682‐4690. 22912387 10.1158/1078-0432.CCR-11-3260

[6] Yan L , Tongtong Z , Jing Z , et al. Response to crizotinib in advanced ALK‐rearranged non‐small cell lung cancers with different ALK‐fusion variants. Lung Cancer. 2018;118:128‐133. 29571990 10.1016/j.lungcan.2018.01.026

[7] Ross CD , Rafal D , Solange P , et al. Updated efficacy and safety data and impact of the EML4‐ALK fusion variant on the efficacy of Alectinib in untreated ALK‐positive advanced non‐small cell lung cancer in the global phase III ALEX study. J Thorac Oncol. 2019;14:1233‐1243. 30902613 10.1016/j.jtho.2019.03.007

[8] Childress Merrida A , Himmelberg Stephen M , Huiqin C , et al. ALK fusion partners impact response to ALK inhibition: differential effects on sensitivity, cellular phenotypes, and biochemical properties. Mol Cancer Res. 2018;16:1724‐1736. 30002191 10.1158/1541-7786.MCR-18-0171 PMC6214753

[9] Yosuke A , Rie I , Toshio S , et al. Oncogenic TPM3‐ALK activation requires dimerization through the coiled‐coil structure of TPM3. Biochem Biophys Res Commun. 2015;457:457‐460. 25596129 10.1016/j.bbrc.2015.01.014

